# Misonidazole enhancement of acute and late radiation injury to the rat spinal cord.

**DOI:** 10.1038/bjc.1979.153

**Published:** 1979-07

**Authors:** J. M. Yuhas


*Br. J. Cancer* (1979) **40**, 161

***Short Communication***

### MISONIDAZOLE ENHANCEMENT OF ACUTE AND LATE RADIATION INJURY TO THE RAT SPINAL CORD

J. M. YUHAS

*From the Cancer Research and Treatment Center, and Department of Radiology, University of New Mexico, Albuquerque, New Mexico, U.S.A.*

Received 13 February 1979 Accepted 14 March 1979

FOR HYPOXIC CELL SENSITIZERS to offer a therapeutic gain in solid-tumour radiotherapy, their toxic effects, either alone or in combination with radiation, have to be expressed selectively in the host's tumour but not in normal tissues. It is already clear that these drugs can radiosensitize and possibly be toxic to solid tumours in the laboratory and the clinic, but what is not clear is whether these effects are expressed selectively in the tumour tissues. Under appropriate experimental conditions, it has been possible to demonstrate radiosensitization of the murine skin (Brown, 1975; Yuhas *et al.*, 1977) and testis (Suzuki *et al.*, 1977) after injection of misonidazole (Ro-07-0582, MIS). One could argue logically that these observations would not limit the clinical application of MIS because the tissues involved are seldom radiation-dose limiting, and the demonstration of radiosensitization required single large doses of radiation which are far beyond those used clinically. These data do suggest, however, that normal tissues contain an as yet unknown fraction of hypoxic cells, or that the action of this drug is more complicated than is realized at present.

Whatever the basis of these observations, we can no longer assume that normal tissues will be totally unresponsive to the toxic and radiosensitizing actions of MIS under all conditions. This conclusion is unsettling, especially when one considers two facts: systems which are sensitive to the toxic effects of MIS are also susceptible to its radiosensitizing activity, and clinical studies with MIS have shown a significant incidence of drug-induced peripheral neuropathies (Disch *et al.*, 1978). These facts, taken together, suggested to us that MIS might radiosensitize neural tissue in general, and the spinal cord in particular.

To test this possibility we chose the X-ray-induced spinal-cord-paralysis system in the Fischer 344 rat. In performing these studies, however, an acute reaction was detected which is summarized in the Table. No acute mortality (death within 10 days) was observed when the rats were given 750 mg/kg of MIS or when they were given 1400–3000 rad of X-rays to a 16mm segment of the lumbar spinal cord. When an injection of 750 mg/kg of the drug was followed 45 min later by the localized radiation, a statistically significant increase in the number of deaths occurring within 24 h was found (Table ). Administration of the treatments in the reverse order produced no mortality, suggesting either that the interaction resulted from radiosensitization or that the drug, when administered after exposure, did not reach the irradiated cord in time to generate the toxicity.

Although interesting, this acute mortality is difficult to relate to the clinical situation, and we wished to determine whether MIS would alter the resistance of rats to late spinal-cord injury, *i.e.*, paralysis. To avoid the acute toxicity, the drug dose was reduced to 200 mg/kg

TABLE.—*Interaction of misonidazole (MIS) and radiation (X-rays) injury to the spinal cord in producing mortality within 24 h**

| Treatment | 24h Mortality (No. dead/No. treated) |
|---|---|
| Radiation only† | 0/71 |
| 750 mg/kg MIS | 0/12 |
| Both treatments | 9/24‡ |
| 2400 rad | 0/12 |
| MIS (750 mg/kg), 45 min. 2400 rad | 4/12§ |
| 2400 rad, 2–3 min., MIS (750 mg/kg) | 0/12 |
| MIS (750 mg/kg) | 0/12 |
| MIS (200 mg/kg), 45 min, radiation† | 0/51 |

* All deaths occurred within 24 h after exposure; survivors are at present being followed for analysis of late paralysis.

† 1400–3000 rad to the spinal cord; details of exposure as in Figure.

‡ $P<0{\cdot}001$ relative to X-rays only; $P<0{\cdot}025$ relative to drug only.

§ $P<0{\cdot}05$ relative to X-rays only, drug only, and drug after X-rays.

which, as shown in the Table, produces no acute deaths, and 45 min after injection the rats were given graded doses of localized X-rays as above.

The Figure summarizes the results of this pilot study through 9·5 months from exposure, and shows that MIS sensitized the rats to radiation-induced spinal-cord paralysis. The radiation doses required to produce paralysis in 50% of the irradiated rats (Finney, 1962) were 2246 rad in control rats and 1744 rad in MIS-injected rats. Depending on whether one includes or excludes the high-dose control groups and the assumptions made in significance testing, this sensitizer enhancement ratio of 1·28 (2246/1744) just achieves or just misses statistical significance at the 5% probability level. Since these data are preliminary, and the raw data can be gleaned readily from the Figure, we leave the question of statistical significance open. Also included in the Figure are data for rats given the radioprotectant WR-2721 (200 mg/kg, 15 min before exposure) under the same conditions. As would be predicted from other analyses on the ability of this

FIG.—Percent of Fischer 344 rats paralysed at 9·5 months after exposure, as a function of X-ray dose. Female rats (90–100 days old) were exposed on a 300 kVp X-ray unit at a dose rate of 196 rad/min. The exposed segment of the spinal cord measured 16 mm and extended from L1 down to and including L4/5. Rats were given an i.p. injection of 200 mg/kg of misonidazole (X) 45 min before exposure, 200 mg/kg of WR-2721 (Δ) 15 min before exposure, or an injection of saline (●). Each point is based on findings in 5 rats and all rats received an i.m. injection of fentanyl (0·08 mg) plus droperidol (0·4 mg) 10 min before exposure. Incidence of paralysis is significantly greater by $\chi^2$ analysis in the MIS-treated rats than in the saline-treated controls at both the 1800 rad ($P<0{\cdot}05$) and 2200 rad ($P<0{\cdot}01$) dose levels. The $LD_{50}$ for MIS in these rats lies between 1000 and 1100 mg/kg.

drug to protect the central nervous system (Yuhas and Storer, 1969) no protection was apparent (Fig.).

Two points need to be considered in these studies: whether the anaesthetic used (see legend to Fig.) contributed to the sensitization by interacting with MIS directly or, more likely, indirectly, and whether the apparent sensitization represents an accelerated rate of appearance of injury, as opposed to true sensitization. We have not yet been able to design a holding device which produces reliable positioning of the unanaesthetized rat without introducing the possibility of physiological stress, so the question of anaesthetic interaction remains open. It would not appear likely, however, that the apparent sensitization is merely accelerated appearance of injury. Paralysis began to appear at 5·0–5·5 months and has reached plateaux at the levels described in the Figure over the past 2 months. Unless a new wave of paralysis appears in the future, it would appear that true sensitization is the underlying mechanism.

Although these data are limited, they do indicate that radiosensitization of the spinal cord, and possibly other neural elements, needs to be considered in using MIS in combination with radiotherapy. We report these data at this preliminary stage as a precautionary note, since the use of MIS in clinical radiotherapy is continually expanding (Disch *et al.*, 1978). Further experiments are at present in progress to determine whether this radiosensitization persists under conditions comparable to those in the clinic.

### REFERENCES

BROWN, J. M. (1975) Selective radiosensitization of the hypoxic cells of mouse tumors with nitroimidazoles, metronidazole and Ro-07-0582. *Radiat. Res.*, **64,** 633.

DISCH, S., SAUNDERS, M. I., ANDERSON, P. & 6 others (1978) The neurotoxicity of misonidazole: pooling of data from 5 centres. *Br. J. Radiol.*, **51,** 1023.

FINNEY, D. J. (1962) *Probit Analysis—A Statistical Treatment of the Sigmoid Dose Response Curve.* Cambridge: University Press. p. 318.

SUZUKI, N., WITHERS, H. R. & HUNTER, N. (1977) Radiosensitization of mouse spermatogenic stem cells by Ro-07-0582. *Radiat. Res.*, **69,** 598.

YUHAS, J. M. & STORER, J. B. (1969) Chemoprotection against three modes of radiation death in the mouse. *Int. J. Radiat., Biol.*, **15,** 233.

YUHAS, J. M., YURCONIC, M., KLIGERMAN, M. M., WEST, G. & PETERSON, D. F. (1977) Combined use of radioprotective and radiosensitizing drugs in experimental radiotherapy. *Radiat. Res.*, **70,** 433.


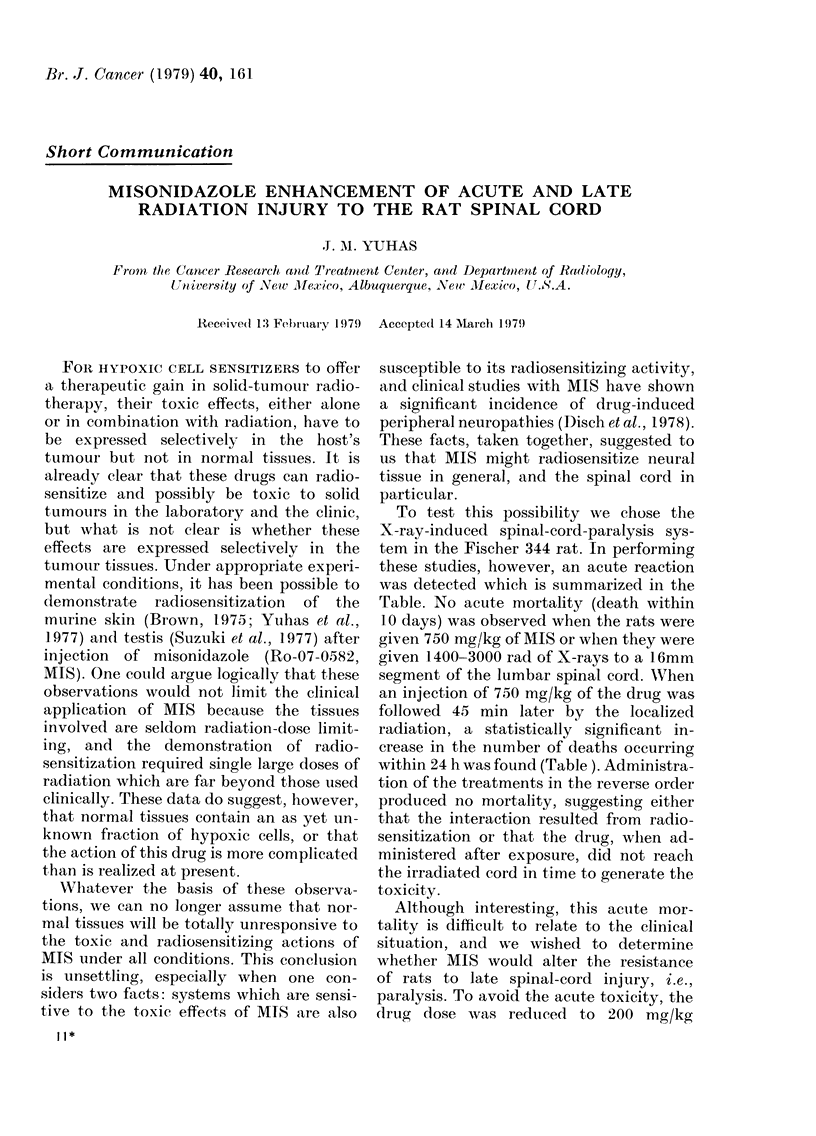

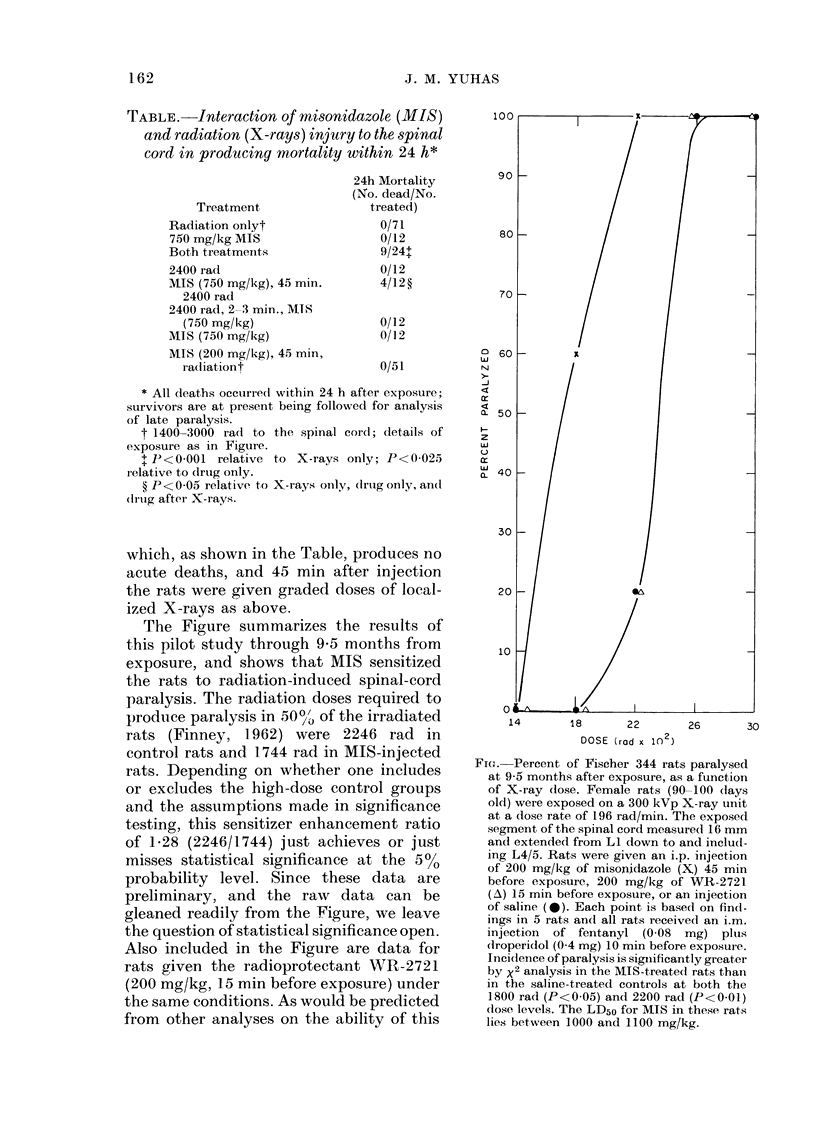

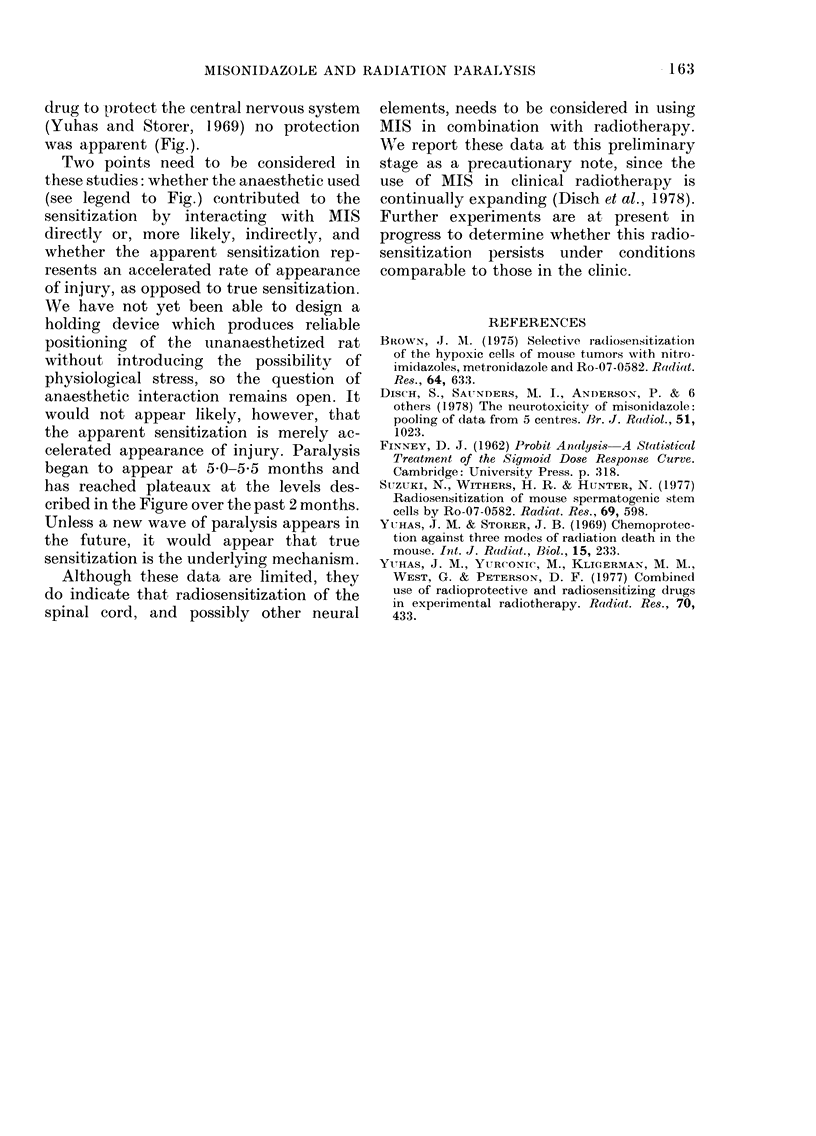


## References

[OCR_00285] Brown J. M. (1975). Selective radiosensitization of the hypoxic cells of mouse tumors with the nitroimidazoles metronidazole and Ro 7-0582.. Radiat Res.

[OCR_00289] Disch S., Saunders M. I., Anderson P., Urtasun R. C., Karcher K. H., Kogelnik H. D., Bleehen N., Phillips T. L., Wasserman T. H. (1978). The neurotoxicity of misonidazole: pooling of data from five centres.. Br J Radiol.

[OCR_00300] Suzuki N., Withers H. R., Hunter N. (1977). Radiosensitization of mouse spermatogenic stem cells by Ro-07-0582.. Radiat Res.

[OCR_00305] Yuhas J. M., Storer J. B. (1969). Chemoprotection against three modes of radiation death in the mouse.. Int J Radiat Biol Relat Stud Phys Chem Med.

[OCR_00310] Yuhas J. M., Yurconic M., Kligerman M. M., West G., Peterson D. F. (1977). Combined use of radioprotective and radiosensitizing drugs in experimental radiotherapy.. Radiat Res.

